# Surface degradation of glass ceramics after exposure to acidulated phosphate fluoride

**DOI:** 10.1590/S1678-77572010000200010

**Published:** 2010

**Authors:** Vanessa Zulema S. CCAHUANA, Mutlu ÖZCAN, Alfredo Mikail Melo MESQUITA, Renato Sussumo NISHIOKA, Estevão Tomomitsu KIMPARA, Marco Antonio BOTTINO

**Affiliations:** 1 DDS, MSc, PhD student, São José dos Campos Dental School, Department of Dental Materials and Prosthodontics, São Paulo State University, São José dos Campos, Brazil.; 2 Dr. Med. Dent., PhD, Professor, University of Zürich, Head of Dental Materials Unit, Center for Dental and Oral Medicine, Clinic for Fixed and Removable Prosthodontics and Dental Materials Science, Zürich, Switzerland.; 3 DDS, MSc, PhD, Lecturer, São José dos Campos Dental School, Department of Dental Materials and Prosthodontics, São Paulo State University, São José dos Campos, Brazil.; 4 DDS, MSc, PhD, Professor and Chair, São José dos Campos Dental School, Department of Dental Materials and Prosthodontics, São Paulo State University, São José dos Campos, Brazil.

**Keywords:** Acidulated phosphate fluoride, Dental ceramics, Dental materials, Loss mass analysis, Microscopy, electron, scanning, Surface roughness

## Abstract

**Objective:**

This study evaluated the surface degradation effect of acidulated phosphate fluoride (APF) gel exposure on the glassy matrix ceramics as a function of time.

**Material and methods:**

Disc-shaped ceramic specimens (N = 120, 10/per ceramic material) were prepared in stainless steel molds (inner diameter: 5 mm, height: 2 mm) using 6 dental ceramics: 3 indicated for ceramic-fused-to-metal (Vita Omega 900, Carmen and Vita Titankeramik), 2 for all-ceramic (Vitadur Alpha and Finesse^®^ Low Fusing) and 1 for both types of restorations (IPS d.SIGN). The specimens were wet ground finished, ultrasonically cleaned and auto-glazed. All specimens were subjected to calculation of percentage of mass loss, surface roughness analysis and topographical description by scanning electron microscopy (SEM) before (0 min) and after exposure to 1.23 % APF gel for 4 min and 60 min representing short- and long-term etching effect, respectively. The data were analyzed using two-way ANOVA with repeated measures and Tukey`s test (α=0.05).

**Results:**

Significant effect of the type of the ceramics (p=0.0000, p=0.0031) and exposure time (p=0.0000) was observed in both surface roughness and percentage of mass loss values, respectively. The interaction factor between both parameters was also significant for both parameters (p=0.0904, p=0.0258). Both 4 min (0.44±0.1 - 0.81±0.2 µm) and 60 min (0.66±0.1 - 1.04±0.3 µm) APF gel exposure created significantly more surface roughness for all groups when compared to the control groups (0.33±0.2 - 0.68±0.2 µm) (p<0.05). There were no significant differences in percentage of mass loss between the ceramics at 4 min (p>0.05) but at 60 min exposure, IPS d.SIGN showed the highest percentage of mass loss (0.1151±0.11). The mean surface roughness for Vita Titankeramik (0.84±0.2 µm) and Finesse^®^ Low Fusing (0.74.±0.2 µm) was significantly higher than those of the other ceramics (0.59±0.1 µm - 0.49±0.1 µm) and Vita Titankeramik (p<0.05) regardless of the exposure time. A positive correlation was found between surface roughness and percentage of mass loss for all ceramic materials [(r=0.518 (Vitadur Alpha), r=0.405 (Vita Omega 900), r=0.580 (Carmen), r=0.687 (IPS d.SIGN), r=0.442 (Finesse^®^ Low Fusing), r=0.572 (Vita Titankeramik), Pearson`s correlation coefficient)]. The qualitative SEM analysis showed evidence of corrosive attack on all of ceramics at varying degrees.

**Conclusions:**

The ceramics indicated for either metal-ceramic or all-ceramic restorations were all vulnerable to surface texture changes and mass loss after short-term and long-term APF gel exposure.

## INTRODUCTION

Dental ceramics provide similar optical properties with the natural tooth substance, present chemical stability, good physical and mechanical properties, and they have excellent biocompatibility to soft tissues with low plaque adhesion^1,2,18^. The feldspathic ceramics are the conventional ceramic materials for metal-ceramic restorations with the basic composition of a mixture of feldspar and quartz^6^. Such ceramics are high temperature-fused materials based on the basic SiO_2_ that acts as the glassy matrix. Oxides of potassium, sodium, aluminum and boron are so called glass modifiers that are added to the compound in order to decrease the melting temperature by reducing the amount of crosslinking between the oxygen and the glass forming element, silica. However, when they are used in excessive amounts, chemical durability of the ceramic is decreased and it also makes the ceramic more prone to devitrification^1,213,19^. Controlled use of these oxides is necessary in order to attain the desirable properties such as resistance to pyroplastic deformation, glaze, to maintain hardness, chemical stability and fusing at low temperatures^2,13,17,18^.

Based on the sintering temperature, dental ceramics are traditionally classified as high-, medium-, low- and ultra low-fusing ceramics. In general, the high-fusing feldspathic ceramics are more corrosion resistant than ceramics with lower sintering temperature. However, all low-fusing ceramics per se are more corrosion-prone than high-fusing ceramics^20^. Glass ceramics used in dentistry are polycrystalline ceramics that are produced under controlled crystallization process. They are characterized by a feldspar glassy matrix in which several crystalline phases such as alumina, tetracyclicfluoromica, leucite, myca crystals with β spodumene crystals are interspersed^19,20^. Dental glasses are amorphous, non-crystalline and ultra-low fusing ceramic materials intended for veneering of metal or ceramic substructures. Recently, research on ceramics has concentrated on developing a fundamental understanding of ceramic damage as influenced by microstructure^8,9^.

The ultra-low fusing ceramics have been developed to be used with titanium and gold alloys^13,17^. Although, high- and medium-fused ceramics exhibit better corrosion resistance than low- and ultra-low fused ceramics, they are reported to create more wear of the antagonist^1,2^. Some low-fusing ceramics demonstrated less wear of the enamel than conventional feldspathic ceramics^6^. The low-fused ceramics also show higher solubility in water in contrast to mediumfused ceramic^13,17,19^. Variations in the composition and processing techniques could influence their hydrolytic stability and also other environmental conditions may impair their resistance to surface and bulk corrosion^8,9^.

Dental ceramics are affected from stress, dynamic fatigue and degradation of the surface that may in turn influence their physical and mechanical properties. When the ceramics are placed in an aqueous environment, exposure to the chemical solutions, water and other fluids may create microcracks and they start to add damaging mechanical properties^4,8,10,18-21^. Subsequently, this process changes the surface hardness and surface properties promoting plaque accumulation, wear of antagonistic structures and sometimes it may impair the aesthetics especially in the anterior region^3,15-17^. Not only the oral environment but also some prevention media could create damage to the ceramics. Professional fluoride applications are recommended for patients with high caries risks every three months or daily topically in toothpastes or in other forms in order to prevent caries. Acidulated phosphate fluoride (APF) at different concentrations was shown to etch dental composites, porcelain, amalgam and dental cements *in vitro*^4,5,7,12^. Although, recent ceramics present different compositions with more chemical stability and eventually better mechanical behavior, the addition of smaller glass particles can be expected to improve their degradation level under APF gel application. It can be anticipated that the cumulative effect of etching media in contact with the ceramics may lead to surface changes. However, the degree of such damage remains to be investigated representing the worst-case scenario and the results need to be compared to the minimum required time for their application. The goal of this study was to evaluate the degradation of several glass ceramics exposed to APF gel at different durations. The null hypotheses tested were that all glassy matrix ceramics present similar degradation when exposed to APF gel and the application time increases the degradation.

## MATERIAL AND METHODS

Ceramic materials with different compositions and microstructures were selected for the experiments (Figure 1). Ceramic discs (N = 120, 10/per ceramic material) were fabricated according to each manufacturer’s recommendations as described in Table 1 using a stainless steel mold with an internal diameter of 5 mm and height of 2 mm. Ceramic liquid and powder were mixed until a creamy consistency was achieved and excess liquid was blot dried with clean tissue papers (Kimwipes^®^, Lite 200, Kimberly, USA). Ceramic masses of 5 discs at a time were fired in a ceramic oven (Vacumat 40 Vita, Vita-Zahnfabrik, Bad Säckingen, Germany) at the temperatures recommended by the manufacturers. After the sintering process, the specimens were cooled down for 10 min and the surfaces were ground finished with silicone carbide papers up to 600-grit on a rotating disc at 150 cycles/min under water cooling. Test specimens were then ultrasonically cleaned in distilled water for 10 min (Vitasonic II, Vita-Zahnfabrik), air-dried and then auto-glazed following the firing procedures for each ceramic type.

**Figure 1:** Brand names, indications, compositions and manufacturers of the dental ceramics used in this study

**Brand name**	**Indication**	**Ceramic Type**	**Manufacturer**
Vitadur Alpha	All-ceramic	Feldspathic ceramic	Vita Zahnfabrik Bad Saeckingen, Germany
Vita Omega 900	Metal-ceramic	Feldspathic ceramic	Vita Zahnfabrik Bad Saeckingen, Germany
Carmen	Metal-ceramic	Feldspathic ceramic with leucite particles	Dentaurum, Ispringen, Germany
IPS d.SIGN	All-ceramic/metal-ceramic	Low-fusing ceramic with 65% glass, fluorapatite crystals and leucite	Ivoclar Vivadent, Liechtenstein Schaan,
Finesse® Low Fusing	All-ceramic	Ultra-low fusing with 7% leucite microparticles	Dentsply Ceramco, York, USA
Vita Titankeramik	Metal-ceramic	Ultra-low fusing ceramic	Vita Zahnfabrik Bad Saeckingen, Germany

**Table 1:** Firing procedures of the dental ceramics tested

**Ceramics**	**Type of firing**	**Starting Temperature (ºC)**	**Drying Time (min)**	**Final Temperature (ºC)**
Vitadur Alpha	Dentine	600	6	960
	Glaze	600	0	940
Vita Omega 900	Dentine	600	6	900
	Glaze	600	0	900
Carmen	Dentine	400	8	870
	Glaze	500	4	880
IPS d.SIGN	Dentine	870	6	869
	Glaze	870	4	869
Finesse® Low	Dentine	450	5	760
Fusing	Glaze	450	3	750
Vita Titankeramik	Dentine	400	6	770
	Glaze	400	0	770

After auto-glazing, the specimens were subjected to 1.23% APF gel (Nupro AFP, Dentsply, Petropolis, RJ, Brazil) for 4 min and 60 min and rinsed and dried thoroughly, where the first is the recommended duration for clinical topical fluoride application by the manufacturer and the latter represents the extended application duration or the worst-case scenario. Application of the APF gel was achieved in one coat only, using a new disposable brush for each specimen assuring that there were no air bubbles entrapped. The gel was applied in one direction on the specimens by the same operator.

All specimens were evaluated before and after to the APF gel exposure using the following methods:

### Percentage of Mass Loss

The specimens were weighed in a digital scale with an accuracy of 0.1 mg (Mettler Toledo, Columbus, OH, USA) in order to calculate the mass before and after APF exposure using the following equation: [W1 - W2 / W1] x 100 where W1 was considered as the specimen weight before APF gel exposure and W2, the weight after APF exposure^14^.

### Surface Roughness Analysis

The surface roughness (Ra) of the specimens was measured by one operator randomly using a surface profilometer (Hommel-Tester, T200, Schwenningen, Germany). The specimens were placed in fixed table, where the analyzing stylus traced 2 mm length at a speed of 0.1 mm/s. The mean roughness value was calculated from 3 single measurements. Each value represented the distance between the lowest and the highest point of the profile.

### Topographical Analysis

The surfaces of the ceramic specimens to be evaluated were cleaned ultrasonically in 99.9% ethanol at 35 kHz for 10 min. Then the specimens were mounted on aluminum stubs and coated with Au-Pd, resulting in a thin layer of about 100300 nm. The topographical analysis of the specimens was made with a a scanning electron microscope (JEOL, JSM-5310 LV, CTA, Tokyo, Japan) at x500 and x5,000 magnifications.

### Statistical Analysis

The results were analyzed using two-way analysis of variance (ANOVA) with repeated measures and multiple comparisons were made using Tukey’s test at a confidence level of 95%. The correlation between surface roughness and loss mass percentage was investigated using Pearson’s correlation test (*p*<0.01).

## RESULTS

Significant effect of the type of the ceramics (*p*=0.0000, *p*=0.0031) and exposure time (*p*=0.0000) was observed for both surface roughness and percentage of mass loss values, respectively. The interaction factor between both parameters was also significant for both parameters (*p*=0.0904, *p*=0.0258) (Tables 2 and 3).

**Table 2:** Results of 2-way analysis of variance for the surface roughness measurements, ceramic types and the interaction terms after different APF exposure times (* *p* < 0.05)

**Effect**	**DF**	**SS**	**MS**	**F**	**P**
Type of ceramic	5	3.0020	0.6004	16.56	0.0000*
Exposure time	2	3.6995	1.8497	91.86	0.0000*
Ceramic versus exposure time	10	0.3418	0.0341	1.70	0.0904

**Table 3:** Results of 2-way analysis of variance for the percentage of mass loss measurements, ceramic types and the interaction terms after different APF exposure times (*p < 0.05)

**Effect**	**DF**	**SS**	**MS**	**F**	**P**
Type of ceramic	5	0.03431	0.0068	4.11	0.0031*
Exposure time	1	0.04345	0.0434	24.73	0.0000*
Ceramic versus exposure time	5	0.02454	0.0049	2.79	0.0258*

### Surface Roughness Analysis

Both 4 min (0.44±0.1 - 0.81±0.2 µm) and 60 min (0.66±0.1 - 1.04±0.3 µm) APF gel exposure created significantly more surface roughness for all the groups when compared to the control groups (0.33±0.2 - 0.68±0.2 µm) (*p*<0.05) (Table 4)

**Table 4:** The mean ± standard deviations surface roughness values (μm) for the ceramics before (Control-0 min) and after 4 min and 60 min APF exposure. The same superscripted letters indicate no significant differences (Tukey's test, *p* < 0.05)

		**Surface roughness values (Ra) (μm)**		
**Material**	**0 min**	**4 min**	**60 min**	**Mean**
Vita Titankeramik	0.68±0.16	0.81±0.16	1.02±0.15	0.8389ª
Finesse® Low Fusing	0.53±0.15	0.65±0.16	1.04±0.33	0.7418ª
IPS d.SIGN	0.49±0.15	0.54±0.15	0.72±0.10	0.5878^b^
Vita Omega 900	0.34±0.22	0.55±0.16	0.69±0.12	0.5298^b^
Vitadur Alpha	0.39±0.08	0.44±0.06	0.70±0.14	0.5123^b^
Carmen	0.33±0.15	0.48±0.08	0.66±0.06	0.4911^b^

The mean surface roughness for Vita Titankeramik (0.84±0.2 µm) and Finesse® Low Fusing (0.74.±0.2 µm) were significantly higher (*p*<0.05) than those of the other ceramics (0.59±0.1 µm - 0.49±0.1 µm) regardless of the exposure time (Table 4).

### Percentage of Mass Loss

There were no significant differences in percentage of mass loss between the ceramics at 4 min (p>0.05) but at 60 min exposure, IPS d.SIGN showed the highest percentage of mass loss (0.1151±0.11) (Table 5).

**Table 5:** The mean ± standard deviations percentage of mass loss for the ceramics before (Control-0 min) and after 4 min and 60 min APF exposure. The same superscripted letters indicate no significant differences (Tukey's test, *p* < 0.05)

		**Percentage of mass loss**	
**Material**	**0 min**	** 4 min**	**60 min**
IPS d.SIGN	0.0167±0.03	0.0167±0.03^b^	0.1151±0.11ª
Finesse^®^Low Fusing	0.0226±0.02	0.0227±0.02^b^	0.0559±0.02^ab^
Vitadur Alpha	0.0118±0.03	0.0121±0.03a^b^	0.0556±0.04^b^
Carmen	0.0300±0.02	0.0308±0.02^b^	0.0486±0.02^b^
Vita Omega 900	0.0114±0.02	0.0114±0.02^b^	0.0296±0.03^b^
Vita Titankeramik	0.0033±0.01	0.0037±0.01^b^	0.0212±0.01^b^

There was a positive correlation between surface roughness and percentage of mass loss for all ceramic materials [(r=0.518 (Vitadur Alpha), r=0.405 (Vita Omega 900), r=0.580 (Carmen), r=0.687 (IPS d.SIGN), r=0.442 (Finesse^®^ Low Fusing), r=0.572 (Vita Titankeramik), Pearson’s correlation coefficient)] (Table 6).

**Table 6:** Pearson's correlation coefficient between roughness and percentage of mass loss for the ceramics. *Correlation is significant at * p* < 0.01

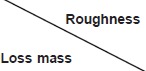	**IPS ** **d.Sign**	**Carmen**	**Vita** ** Titankeramik**	**Vitadur** ** Alpha**	**Finesse® Low** ** Fusing**	**Vita** ** Omega 900**
IPS d.Sign	0.687*					
Carmen		0.580*				
Vita Titankeramik			0.572*			
Vitadur Alpha				0.518*		
Finesse® Low Fusing					0.442*	
Vita Omega 900						0.405*

### Topographical Analysis

**Figure 2:**
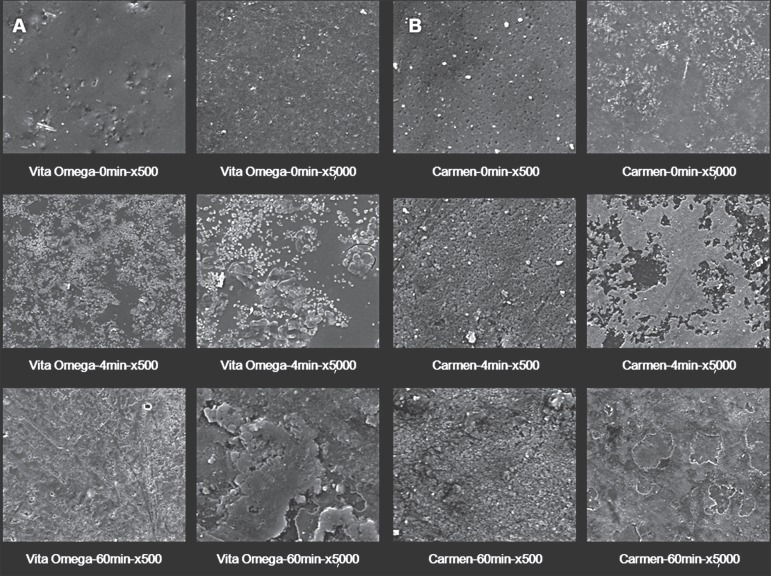
Representative SEM micrographs of A) Vita Omega and B) Carmen at 0, 4 and 60 min time points of APF gel exposure (x500 and x5,000 magnifications). The AFP gel exposure produced mostly linear defects or grooves by attacking the leucite-induced cracks, and phase boundaries. The AFP gel also seems to build up surface deposits preferentially on the leucite crystal phase

**Figure 3:**
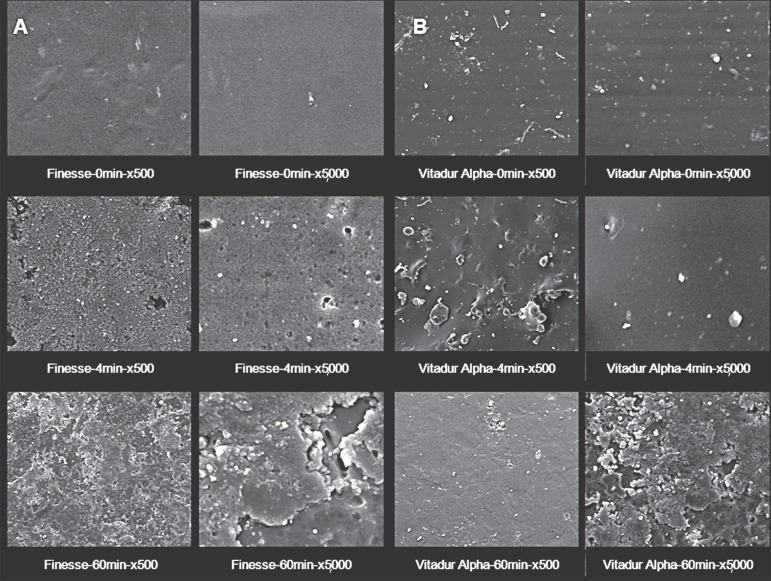
Representative SEM micrographs of A) Finesse and B) Vitadur Alpha at 0, 4 and 60 min time points of APF gel exposure (x500 and x5,000 magnification). A clear trend to rougher surfaces was observed as a function of exposure time. Note also precipitates on the surfaces at 60 min (x5,000)

**Figure 4:**
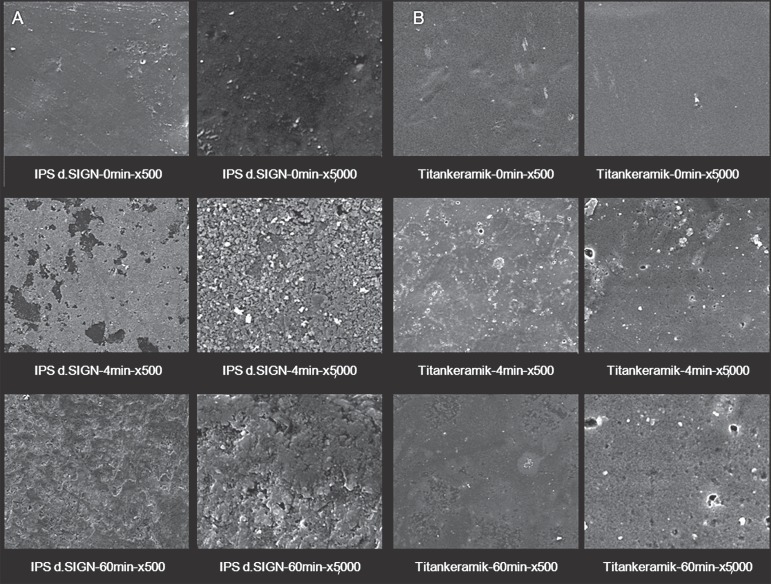
Representative SEM micrographs of A) IPS d.SIGN and B) Vita Titankeramik at 0, 4 and 60 min time points of APF gel exposure (x500 and x5,000 magnification). Note that the 60 min exposure showed an aggressive effect on the surface of the two ceramics, but this effect was less evident for single-phase high crystalline content Vita Titankeramik

The qualitative description of the SEM analysis showed apparent evidence at varying degrees of surface alterations with irregularities characterized with the presence of pores (Figures 2a-b, 3a-b, 4a-b). SEM analysis further verified that the layer of vitrification presented itself with surface characteristics with minimum defects. Such surface patterns were more evident in the ceramics with leucite particles in their compositions.

SEM micrographs displayed surfaces with deposits of particles in the form of precipitate or degradation material in the vitreous matrix.

In general, corrosive attack of APF gel was more evident when the ceramic materials were exposed to this medium for 60 min.

## DISCUSSION

Since both the ceramic type and the application duration affected the results, the null hypotheses tested that all glassy matrix ceramics would present similar degradation when exposed to APF gel is rejected. On the other hand, 4 min application duration did not affect the percentage of mass loss but 60 min affected IPS d.SIGN the most with significant interaction terms. Therefore the second hypothesis is partially accepted.

Topical APF gel application is absolute for reduction of the incidence and progress of the caries. For this reason, this method is utilized by the majority of the dental professionals. However, the presence of existing restorations and their sensitivity to this prophylactic medium is often overlooked by the clinicians^5,12^. Color change that may lead to restoration renewal in the long- term in ceramic restorations also indicates that the ceramic materials are not always stable. In the aggressive oral environment, fatigue forces, temperature and pH changes are a few factors that can affect the integrity of the ceramic materials. Features such as chemical stability, resistance to surface texture changes, and amount of degradation of the ceramics in the oral cavity can show variations depending on the chemical composition and fabrication process of the individual ceramic material.

Ceramic restorations are subject to cyclic loads in the humid oral environment that create ideal conditions for the increase of the existing defects in the form of slow crack growth that contribute to the severe decrease in the durability of the ceramic restorations. Furthermore, this process can be the main factor for the increase in surface hardness when ceramics are exposed to fluids, saliva, water and other chemical substances in the oral environment^17,19^. Ceramic materials are weak in tension and the dynamic fatigue and surface degradation can influence mechanical properties negatively^3^. When ceramic restorations do not maintain their smooth surfaces, this could lead to retention of dental plaque^16^, abrasion of the antagonist teeth^1^, color change^13,14^, injury of the adjacent soft tissues and sometimes improper aesthetic qualities of the restorations^2,6,16^. Therefore surface roughness is an important parameter when evaluating the performance of dental ceramics as well as other dental materials.

In the dental literature, the most frequently used parameter for the surface quality assessment of the dental material is the overall surface roughness, namely Ra values. The stylus traces a given length on a certain surface area offering a quantitative result^1,15^. However surface roughness results are often verified with SEM images in order to assess the surface topography specifically^15,20^. The SEM images demonstrate the shape and contour changes that the surface profilometer may not show^15^. The validity of the contact stylus tracing in surface roughness measurement may sometimes be questionable since degradation is a phenomena that works as a function of time. Depending on the duration of the exposure to the degrading material or medium, a rough surface may result in a smoother texture. Therefore, due to the limitations of the surface roughness measurement methods, the studied surface should be evaluated both quantitatively and qualitatively.

*In vitro* studies have reported surface degradation at pH levels under 3.7^1,18^. In these circumstances, any restorative dental material can be affected and eventually loose its mechanical properties in the acidic environment. However, this was not widely studied in the dental literature^14,18-21^. The 1.23% APF gel studied consists of 2% sodium fluoride, 0.34% of hydrofluoric acid and 0.98% of phosphoric acid that provides a pH of 3.6-3.9^5,7,8,12^. The presence of hydrofluoric acid in the APF gel results in dissolution of the silica, forming a precipitate on the surface thereby generating loss of mass and increased surface roughness^7,9^. It was also suggested that the process of degradation happens due to exchange of alkaline ions. This kind of ion exchange, depending on the composition of the ceramic, could take place at levels of pH below 7^18^. The degradation of the surface occurs mainly either in the areas that consists defects or within different phases of the ceramic materials. The ceramics with elevated content of crystals are attacked in the surface in different forms than the ceramics with few crystals^20^. However, in addition to the effects of composition, microstructure and environmental conditions, surface corroded layer may also influence the degree of degradation^17^ that should be taken into consideration in future studies.

The crystalline phase of the ceramics acts like a nucleus capable to resist or inhibit the crack propagation. Moreover, the form and the size of the particles of the ceramic powder determines the efficacy of the condensation and shrinkage during firing process^4,9,20^. The feldspathic ceramics are composed of a vitreous matrix with different volumes of leucite or alumina and therefore in the presence of a heterogeneous microstructure, the surface of degradation is not uniform may result in increased surface tension^8,9^. Despite the attempt to reduce spaces, a residual volumetric porosity of 45% is present during air-firing or vacuum-firing after compaction^20^.

The new ceramic systems have reduced volume of the particles in the form of leucite microcrystals or silicate of alumina as a reinforcement material decreasing this porosity to 30%. The addition of alkali oxides and glass modifiers in the composition of the ceramics that act like substitutes for molecular flow at lower temperatures that eventually decreases the fusion temperature and viscosity of the ceramics. However, a high proportion of these modifiers reduces the hydrolytic resistance of such ceramics^18,19^. According to the results of this study, IPS d.SIGN ceramic presented the highest values of loss of mass. The reason for this could be due to the presence of 65% glass, fluorapatite crystals and leucite in its composition. However, interestingly this ceramic did not present mean surface roughness values significantly different than those of the other ceramics. Therefore the first hypothesis could be only partially accepted. In fact its surface roughness was considerably lower than those of the two ultra-low fusing ceramics. The rather smooth surface after APF gel exposure could indicate either an increase in corrosion resistance or uniform gradual process of corrosion. The latter phenomenon is associated with decrease or loss of peaks and eventually an even loss of volume from the surface. Therefore a smooth surface should not be considered always as good feature after acid exposure since the volume loss would be also one of the determinants of the mechanical strength of the ceramics.

The peculiar levels of irregularities in the surface can be related to the characteristics of the vitrification process that allows small time and temperature variations^2,6,10,18^. Condensation, cooling, multiple firing processes can produce additional leucite and this generally increases the coefficient of thermal expansion of the ceramics that in turn could also result in breach or crack on the surface. However, testing the mechanical properties of the ceramics after APF gel exposure was not within the scope of this study and should be studied further.

Most of the dental ceramics developed for metal ceramic restorations contain leucite as the principal crystalline phase^10^. In this way, the cracks formed during the chemical attack, results in preferential attack of the regions with residual tensions related with the leucite or depending on the collection of particles of leucita. In the case of the Finesse^®^ Low Fusing and IPS d.SIGN ceramics, the irregularities were presented around the crystals that were found in a smaller quantity in the vitreous matrix due to their composition. On the other hand, Finesse^®^ Low Fusing and Vita Titankeramik ceramics, so called ultra low-fused ceramics, showed the highest mean surface roughness. These results were supported by the SEM micrographs of the same materials. It is known that in order to decrease the fusion temperature of ceramics, the chemical composition is altered, particularly in the quantity of glass modifiers that decreases the hydrolytic resistance of the surface^17-20^. This could be a consequence of the vitrification process associated with the firing temperature that promoted a tension in the ceramic surface.

Roughness parameters are calculated using a formula, describing the surface. There are many different roughness parameters in use such R_z_, R_q_, R_k_, R_y_ but R_a_ is the most commonly used parameter^22^. Since these parameters reduce all of the information in a profile to a single number, great care must be taken in applying and interpreting them. In order to make it possible to compare the results with previous studies^3,4,7,11,15^, in this study the most commonly reported R_a _values were used. These results could be coupled with other roughness parameters which could be judged as the limitation of this study. On the other hand, percentage of loss of mass offsets the possible variations between the roughness parameters and evaluates surface damage in a global sense which can be considered as the strength of this study.

The tested durations of APF gel exposure, namely 4 and 60 min could be considered too long. The manufacturer recommends 4 min of APF gel exposure for preventive measures. The results of this study clearly indicate the compulsory use of rubber dam with which the surfaces of the ceramic restorations could be protected when APF gel is utilized for patients with such restorations. Sixty min could still be considered as a cumulative effect of continuous use of home-used topical fluoride gels. In a similar study, Dionysopoulos, Gerasimou and Tolidis^11^ showed that the APF gel has the most damaging effect on glass-ionomer, resin modified glassionomers, compomers and composite resins when compared to NaF gel for home-use fluoride treatment. In that study, authors reported that 24 h of APF gel exposure was an equivalent of 4 min daily use for 1 year. Surface degradation of these dental ceramics also depends on their surface energy and wettability with APF, and surface roughness. Future studies should address these issues.

In summary, clinicians should consider the existing ceramic restorations and the material types used in such restorations during advising prophylactic measures.

## CONCLUSION

From this study the following could be concluded: 1. No difference was found in the percentage of mass loss between the ceramics at 4-min APF gel exposure, however, the lowfusing ceramic with glass, fluorapatite and leucite in its composition (IPS d.SIGN) showed significantly higher percentage of mass loss values at 60 min than the other tested ceramics; 2. The ultra-low fusing ceramics (Finesse^®^ Low Fusing and Vita Titankeramik) showed the highest mean surface roughness values after 4 and 60 min APF exposure time; 3. The qualitative SEM analysis showed surface changes at varying degrees in all ceramics.
